# Prevalence, Associated Factors, and Consequences of Burnout Among Egyptian Physicians During COVID-19 Pandemic

**DOI:** 10.3389/fpubh.2020.590190

**Published:** 2020-12-03

**Authors:** Ahmed Samir Abdelhafiz, Asmaa Ali, Hany H. Ziady, Ayman Mohamed Maaly, Mohamed Alorabi, Eman A. Sultan

**Affiliations:** ^1^Department of Clinical Pathology, National Cancer Institute, Cairo University, Cairo, Egypt; ^2^Department of Chest, Abbassia Chest Hospital, Ministry of Health and Population (MOHP), Cairo, Egypt; ^3^Department of Community Medicine, Faculty of Medicine, Alexandria University, Alexandria, Egypt; ^4^Department of Anaesthesia and Surgical Intensive Care, Faculty of Medicine, Alexandria University, Alexandria, Egypt; ^5^Department of Clinical Oncology, Faculty of Medicine, Ain Shams University, Cairo, Egypt

**Keywords:** burnout syndrome, Egypt, physicians, COVID-19, Occupational Health

## Abstract

**Objectives:** COVID-19 has been recognized as a pandemic by the World Health Organization, and physicians are at the frontline to confront the disease. Burnout syndrome (BOS) is a syndrome resulting from chronic workplace stress that has not been successfully managed. The objective of this study is to evaluate the frequency and associated risk factors of BOS among a sample of Egyptian physicians during the COVID-19 pandemic.

**Methods:** Using Maslach Burnout Inventory Human Services Survey, a cross-sectional electronic survey was conducted to assess BOS among the target group.

**Results:** Two hundred and twenty physicians participated in the study. The frequency of BOS among the research group was 36.36%. The possibility of development of BOS increased two times with the need to buy personal protective equipment (PPE) from participants' own money, with harassment by patients' families, and was less likely to develop in doctors with older age. While male gender was a predictor of depersonalization (DP), female gender showed a significant association with higher emotional exhaustion (EE). Infection or death from COVID-19 among colleagues or relatives showed significant association with elevated EE and lowered personal achievement (PA), respectively.

**Conclusion:** COVID-19 pandemic added new factors to the development of BOS in our research group. Several measures should be taken to support physicians at this stage. These measures include psychological support, organizing work hours, adjusting salaries, and providing personal protective equipment and training on safety measures.

## Introduction

The World Health Organization declared the COVID-19 outbreak a pandemic in March 2020 (1). The disease has since spread rapidly, with millions of documented infections worldwide. The disease spectrum is broad and ranges from asymptomatic to severe illness, the latter may require hospitalization and artificial ventilation (2). The average global mortality rate is 5.7%, with higher deaths among the elderly and individuals with pre-existing medical conditions (3).

A growing number of cases have been reported in Egypt to reach about one hundred and four thousand cases, and about 6,000 deaths by the 1st week of October 2020. Healthcare workers, including physicians, are at the frontline of the battle against this disease. In Egypt, physicians facing the greatest challenges in dealing with the outbreak have been the pulmonologists, anesthesiologists, intensivists, and tropical medicine specialists. Rapid decision making has been the key to proper diagnoses, isolation, and successful treatment of cases. Another obvious source of stress is the increased risk of exposure to COVID-19 among these physicians, raising worries about contracting the disease and/or transmitting it to their families and loved ones. Furthermore, the COVID-19 pandemic, unpreceded in our lifetimes, has caused a growing burden on medical services in both developing and developed countries. This burden, together with the shortage of human and non-human resources resulted in a marked increase in the workload among healthcare workers. All of the above challenges could potentially lead to a rise in mental health problems among physicians and other healthcare workers (4, 5).

Burnout syndrome (BOS) is a syndrome that results from chronic workplace stress that had not been properly addressed. The three dimensions of BOS are (i) feelings of energy depletion or emotional exhaustion and of being “mentally distant” from one's job; (ii) feelings of negativism or cynicism in relation to one's job (depersonalization); and/or (iii) reduced professional achievement (6). The prevalence of BOS among physicians prior to the COVID-19 pandemic had varied between 50 and 70%. Higher levels of BOS were observed among younger physicians and critical medical specialists (7, 8). The objective of the current study was to evaluate the frequency of BOS among a sample of Egyptian physicians due to the COVID-19 pandemic, and to focus on associated risk factors and potential consequences.

## Methods

### Study Design, Target Population, and Sampling

A cross-sectional electronic survey was conducted during the month of April 2020. An online survey form was created using Google Forms, and participants were invited to respond to the questionnaire. Physicians working in Egypt of different specialties, particularly frontline specialists such as pulmonologists, anesthesiologists and intensivists, were invited to participate in the survey.

Convenience sampling through solicitation of survey responses was performed. The sample size was determined using the Epi Info 7 software program based on the expected frequency of burnout syndrome among physicians dealing with COVID-19 cases (13%) (9) at a 95% confidence interval and a limit of 5% precision. Based on these parameters, the calculated sample size was 174 participants.

### Data Collection Tool

The survey questionnaire was divided into three sections: The first section covered socio-demographic characteristics and employment-related information. The second section covered physicians' history of dealing with COVID-19 cases and related deaths as well as their perceptions of the reality of the COVID-19 situation in Egypt. The third section evaluated burnout syndrome using the Maslach Burnout Inventory Human Services Survey (MBI-HSS) after getting the required permission (10). The latter consists of 22 questions that explore the three dimensions of burnout; emotional exhaustion (EE; nine items); depersonalization (DP; five items); and personal achievement (PA; eight items). Each question is rated on a seven-point Likert scale, from never (score 0) to every day (score 6). Scores of all questions within each subscale were added up separately to attain “total dimension scores.” For each responder, the total score of each dimension was classified as low, moderate or high according to the cut-offs displayed in footnote of **Table 2B** (10, 11). A common approach for diagnosis considers respondents with high levels of either EE (Total score of 27 or higher) or DP (Total score of 10 or higher) as having burnout (12). Other researchers have adopted an alternative approach that considers individuals to have a burnout if they had a high EE score plus either a high DP score or a low PA score (PA score < 33) (13). In our study, we used the second approach, as it considers all three dimensions of burnout in coming to diagnosis.

### Statistical Analysis

Statistical analysis was performed using Minitab 17.1.0.0 for Windows (Minitab Inc., 2013, Pennsylvania, USA). Continuous data were presented as means and standard deviations, and categorical data as numbers and percentages. The normality of data was examined using the Kolmogorov Smirnov test. Independent *t*-test and Pearson correlation coefficient test were used to estimate the association/correlation between different demographic characters of participants and some risk factors with BOS domains, while one-way ANOVA test was used to estimate the association between degree of agreement of participants and BOS domains. Multiple logistic regression models with stepwise elimination technique and adjustment for age, sex, comorbidity, and marital status were performed to find any possible predictors for BOS and its three domains. All tests were two-sided, *P* was considered significant if ≤ 0.05.

### Ethical Considerations

This study was approved by the ethics committee of Alexandria University. Measures were taken to ensure respondent anonymity and confidentiality. The submission of the answered survey was considered as the physician giving consent to their participation in the study.

## Results

### Socio-Demographic Data and Employment Characteristics of Participants

Two hundred and twenty medical physicians participated in the study. Most respondents were young physicians (mean age of 33.42 + 5.28 years), with equal participation of both genders. Most of them were married and had children. The largest proportion lived in Cairo and Alexandria. Around half of them had completed master's degrees, and one-tenth had earned doctorates. Among the study sample, 34.09% were pulmonologists and 14.55% were anesthesiologists and intensivists. Other socio-demographic data and job characteristics of participants are presented in Table 1.

**Table 1:** Socio-demographic data and job characteristics of participants.

**Characteristic**	**Total (*****n*** **=** **220)**	
**Age**		
Mean ± SD	33.42 ± 5.28	
	**No**	**%**
**Sex**		
Male	111	50.45
Female	109	49.55
**Marital status**		
Single	58	26.36
Married	151	68.64
Other	11	5.00
**Having children**		
Yes	143	65.00
No	77	35.00
**Have a chronic disease**		
Yes	44	20.00
No	176	80.00
**Smoking habits**		
Smoker	27	12.27
Non smoker	193	87.73
**Residence place**		
Cairo	87	39.55
Alexandria	63	28.64
Delta	39	17.73
Suez Canal	22	10.00
Upper Egypt	9	4.09
**Work place**		
Same as residence area	195	88.64
Other	25	11.36
**Highest degree attained**		
MBBCh	70	31.82
Diploma	21	9.55
Master	107	48.64
Doctorate	22	10.00
**Specialty**		
Chest	75	34.09
Anaesthesia and ICU	32	14.55
Internal medicine	21	9.55
Family medicine	11	5.00
Surgery	11	5.00
Radiology	8	3.64
Tropical medicine	6	2.73
Pediatrics	5	2.27
Emergency medicine	4	1.82
Clinical pathology	3	1.36
Cardiology	3	1.36
Others	41	18.63
**Years of work**		
<5 years	50	22.73
From 5–10 years	87	39.55
From 10–20 years	73	33.18
More than 20 years	10	4.55
**Change in work hours after COVID-19**		
Decreased	109	49.55
Increased	51	23.18
Stable	60	27.27

*Continues data is presented as mean and SD, and categorical data as numbers and percentages.

### Risk Factors and Some Perceptions Related to COVID-19

Supplementary Table 1 details the different COVID-19 risk factors among our participants. About 81% of participants were dealing with COVID-19 patients and/or their samples mainly through their work in triage or isolation hospitals. The majority (73.64%) had purchased PPE on their own expenses. Around one-fourth (27.73%) reported mortality among COVID-19 patients in their workplaces. Treatment protocols and cure rates from COVID-19 were satisfactory for almost two-third of the participants. Despite that, around 65% of them were unsatisfied with the coordination between triage and isolation hospitals. About 40.78% of the participants had experience with harassment from families of COVID-19 patients. In our cohort, only two physicians had COVID-19 infection. Participants reported a high infection rate among their colleagues (59.55%), unlike their relatives (3.64%). Mortality rates among participants' colleagues and relatives were low (4.55%).

Perceptions of participants about COVID-19 in Egypt are summarized in Supplementary Table 2. More than half the sample “agreed” or “totally agreed” that personal protective equipment (PPE) presence in the workplace was satisfactory. However, around one half “disagreed” or “totally disagreed” that disease awareness among the general public was acceptable. Also, more than one third (37.72%) of the sample responded with “disagree” or “strongly disagree” when asked if the general public appreciated efforts made by health professionals to combat COVID-19. Most participants were not satisfied with their salaries. Moreover, the majority “agreed” or “totally agreed” that there was a stigma against both COVID-19 patients and health professionals working with them in Egypt.

### Prevalence of BOS and the Degree of Severity of Affection Within Each Domain

The prevalence of BOS among the sample physicians was 36.36%. The mean values of BOS scale domains were 20.67 + 13.34, 7.20 + 6.14, and 18.53 + 10.19 for EE, DP, and PA, respectively (Table 2A). The severity of different domains of BOS are listed in Table 2B.

**Table 2A:** Mean scores of BOS domains among participants (*n* = 220).

**BOS domain**	**Score level**	
	**Mean**	**SD**
EE	20.67	13.34
DP	7.20	6.14
PA	18.53	10.19

*BOS, Burnout syndrome; EE, emotional exhaustion; DP, Depersonalization; PA, professional achievement.

**Table 2B:** Severity of BOS domains among participants (*n* = 220).

**BOS domain**	**Severity**					
	**Mild**		**Moderate**		**Severe**	
	***N***	**%**	***N***	**%**	***N***	**%**
EE	108	49.09	50	22.73	62	28.18
DP	105	47.73	45	20.45	70	31.82
PA	10	4.55	14	6.36	196	89.09
	**Mean**	**SD**	**Mean**	**SD**	**Mean**	**SD**
EE	8.96	0.454	22.22	0.363	37.89	1.08
DP	2.795	0.188	9.55	0.225	17.632	0.714
PA	43.27	1.02	35.118	0.521	15.646	0.51

*For EE mild level is defined as score between 0 and 18, moderate level between 19 and 26, and high level ≥ 27. For DP low mild is defined as score between 0 and 5, moderate level between 6 and 9, and high level ≥ 10. For PA mild level is defined as score ≥ 40, moderate level between 34 and 39, and high level between 0 and 33.

**BOS, Burnout syndrome; EE, emotional exhaustion; DP, Depersonalization; PA, professional achievement.

### Association Between Demographic Characteristics and Other Risk Factors With the Three BOS Domains

Results suggest that participants' socio-demographic characteristics had influenced the degree of BOS. Table 3 shows that while younger age was significantly correlated with higher EE and DP score (*r* = −0.13, *P* = 0.05 for both), and with lower PA score (*r* = 0.21, *P* = 0.004.), lack of experience was significantly associated with lower PA score (*r* = 0.22, *P* = 0.003). Higher EE scores were significantly associated with female gender (*P* = 0.01), work in isolation hospital ICU (*P* = 0.007), and with infection of a colleague with COVID-19 (*P* = 0.02). Lower PA scores were significantly correlated with dissatisfaction about cure rate of patients (*P* = 0.04), and death of a colleague or relative due to infection with COVID-19 (*P* = 0.04).

**Table 3:** Association between demographic characteristics and other risk factors with different BOS domains.

**Factors**	**EE**			**DP**			**PA**		
	**Mean**	***SD***	***P***	**Mean**	***SD***	***P***	**Mean**	***SD***	***P***
**Sex**									
Male	18.6	13.1	0.01^$^	7.58	6.27	0.35	18	10.5	0.42
Female	22.8	13.3		6.82	6		19.08	9.88	
**Marital status**									
Married	19.9	12.9	0.24	6.8	5.89	0.17	18.33	9.64	0.68
Other	22.3	14.1		8.07	6.61		19	11.4	
**Comorbidity**									
No	20.2	13.3	0.27	7.05	6.12	0.46	18.3	10.2	0.42
Yes	22.7	13.6		7.82	6.25		19.6	10.2	
**Smoking habits**									
Non smoker	20.7	13.1	0.84	7.08	6.02	0.48	18.4	10.1	0.69
Smoker	20.1	14.9		8.07	6.96		19.3	10.9	
**Having children**									
No	21.8	14	0.38	7.96	6.45	0.18	17.9	11.2	0.51
Yes	20.1	13		6.79	5.94		18.87	9.62	
**Work in isolation hospital**									
No	19.7	12.4	0.13	6.84	5.89	0.21	18.2	10.7	0.49
Yes	22.9	15.1		8.01	6.62		19.19	8.87	
**Work in isolation hospital ICU**									
No	19.4	12.6	0.007^$^	6.82	5.9	0.06	18.2	10.3	0.2
Yes	27.1	15.3		9.14	7		20.42	9.42	
**Work in triage hospital**									
No	21.1	13.8	0.53	7.04	5.95	0.6	17.8	9.25	0.17
Yes	19.9	12.4		7.51	6.51		19.9	11.7	
**Buying PPE from his/her own money**									
No	15.1	11.1	<0.001^$^	5.36	5.27	0.004^$^	16.91	9.61	0.14
Yes	22.6	13.5		7.86	6.31		19.1	10.4	
**Watching mortality of patients from COVID-19 at workplace**									
No	20.3	13.2	0.55	6.77	5.77	0.12	18.5	10.3	0.96
Yes	21.5	13.7		8.33	6.93		18.57	9.87	
**Satisfaction with the cure rate of COVID-19 patients**									
No	20.6	12.5	0.97	6.89	5.86	0.31	17.52	9.66	0.04^$^
Yes	20.7	14.9		7.82	6.65		20.6	11	
**Satisfaction with the treatment protocol for COVID-19 patients**									
No	20.9	13	0.69	7.26	6.12	0.81	17.85	9.8	0.13
Yes	20.1	14.2		7.05	6.23		20.2	11	
**Satisfaction with the coordination between triage and isolation hospitals**									
No	20.8	13.5	0.76	7.2	6.13	0.98	17.99	9.53	0.25
Yes	20.2	13		7.19	6.21		20.2	12	
**Harassment by patients' families during work with COVID-19 Patients**									
No	18.9	12.6	<0.001^$^	6.3	5.44	0.001^$^	17.87	9.76	0.09
Yes	27.3	14.2		10.51	7.39		21	11.4	
**Colleagues infected with COVID-19**									
No	18.2	12.7	0.02^$^	6.29	6.06	0.07	17.6	10.5	0.25
Yes	22.4	13.6		7.82	6.14		19.19	9.96	
**Relatives infected with COVID-19**									
No	20.5	13.4	0.35	7.02	6.06	0.09	18.6	10.2	0.84
Yes	24.6	11.4		11.88	6.88		17.88	9.4	
**Colleagues or relatives died from COVID-19**									
No	20.6	13.4	0.57	7.09	6.12	0.27	18.8	10.3	0.04^$^
Yes	23.1	13.4		9.5	6.49		13.2	7.28	
	**r**	**P**		**r**	**P**		**R**	**P**	
Age	−0.13	0.05*		−0.13	0.05*		0.21	0.004*	
Years of work	−0.06	0.35		−0.11	0.10		0.22	0.003*	
Hours of work per week	0.04	0.60		0.06	0.35		0.06	0.394	

* *BOS, burnout syndrome; PPE, Personal protective equipment; EE, emotional exhaustion; DP, Depersonalization; PA, professional achievement*.

$ *Independent t-test, P considered significant if ≤ 0.05*,

** *Pearson correlation coefficient, the sign before “r” denote the direction of relationship, P ≤ 0.05 considered significant*.

Both higher EE and DP scores were associated with the need for buying PPE from private money (*P* < 0.001 and 0.004, respectively), and harassment by patient's families during work (*P* < 0.001 for both).

### Association Between Perceptions of the COVID-19 Situation in Egypt and Scores in the Three BOS Domains

As shown in Table 4, higher EE levels were significantly associated with dissatisfaction regarding the presence of PPE in the workplace (*P* = 0.04), lack of public awareness about the disease (*P* = 0.01), insufficient public appreciation of physicians' work during the pandemic (*P* = 0.01), as well as dissatisfaction about the salary (*P* = 0.02).

**Table 4:** Association between perceptions and different BOS domains.

			**EE**			**DP**			**PA**		
		***N***	**Mean**	***SD***	***P***	**Mean**	***SD***	***P***	**Mean**	***SD***	***P***
Q1	A	114	19.23	13.63	0.04*	7.09	6.17	0.52	19.01	9.89	0.68
	D	65	24.08	12.21		7.85	5.96		18.40	10.41	
	N	41	19.27	13.57		6.49	6.37		17.41	10.80	
Q2	A	36	17.17	9.91	0.01*	6.53	4.81	0.42	18.36	8.62	0.50
	D	131	22.83	13.93		7.65	6.36		19.12	9.96	
	N	53	17.70	12.99		6.55	6.37		17.19	11.69	
Q3	A	59	19.92	13.17	0.01*	7.98	6.53	0.21	19.17	9.96	0.79
	D	83	23.77	13.97		7.53	6.14		18.59	10.36	
	N	78	17.94	12.23		6.26	5.78		17.99	10.28	
Q4	A	34	17.85	12.23	0.02*	6.68	5.95	0.78	18.41	10.90	0.39
	D	154	22.26	13.61		7.39	6.36		19.02	10.24	
	N	32	16.00	11.83		6.84	5.30		16.31	9.11	
Q5	A	178	21.39	13.45	0.25	7.57	6.38	0.15	18.89	10.53	0.20
	D	17	17.18	11.03		6.24	5.03		14.29	6.13	
	N	25	17.92	13.71		5.20	4.58		18.84	9.51	
Q6	A	172	21.35	13.73	0.28	7.31	6.28	0.43	18.84	10.20	0.69
	D	13	20.46	12.08		8.54	6.46		17.54	11.40	
	N	35	17.40	11.55		6.17	5.26		17.37	9.85	

* *A: includes those who agreed and strongly agreed to this question, D: includes those who disagreed and strongly disagreed to this question, N: Not sure. Q1: I feel satisfied with the presence of personal protective equipment where I work, Q2: I think there is an accepted level of awareness about the disease among the public, Q3: I think the general public appreciates the efforts of health professionals against COVID-19, Q4: I feel stratified with the salary I get, Q5: I think there is stigma against patients with COVID-19 in Egypt, Q6: I think there is stigma against health professional dealing with COVID-19 in Egypt*.

** *One-way ANOVA test, P considered significant if ≤ 0.05*.

### Predictors of BOS Among Participants

The logistic regression model was used to identify predictors of BOS among the study group (Table 5). The possibility of development of BOS increased up to two times with the need to buy PPE from participants' own money, and with harassment by patients' families (OR = 2.17 and 2.03, respectively, *P* = 0.04 for both). On the other hand, BOS was less likely to develop in physicians with older age (OR = 0.9; *P* = 0.002).

**Table 5:** Predictors of BOS among participants.

**Factors**	**OR**	**95% CI**	***P***
Age	0.90	(0.8424, 0.9680)	0.002
Sex (male)	1.58	(0.8587, 2.9161)	0.14
Marital status (single)	1.25	(0.4945, 3.1567)	0.64
Having children (yes)	1.48	(0.5766, 3.8136)	0.41
Comorbidity (yes)	1.49	(0.7043, 3.1471)	0.30
Buy PPE from own money (yes)	2.17	(1.0363, 4.5494)	0.04
Watching mortality from COVID-19 in workplace (yes)	1.16	(0.5836, 2.3236)	0.67
Harassment by patients' families during work with COVID-19 Patients (yes)	2.03	(0.9923, 4.1536)	0.04
Colleagues or relatives died from COVID-19 (yes)	1.09	(0.2710, 4.3723)	0.91

*OR, odd ratio; CI, Confidence interval. P value considered significant if < 0.05*.

We then used the same regression model to identify predictors of the different BOS domains. Some of the aforementioned factors were also associated with EE domain of BOS (Supplementary Table 3), where the possibility of getting high EE scores increased up to three times with the need for buy PPE from participants' own money, and two times with harassment by patients' families (OR = 2.73 and 2.21, respectively, *P* = 0.02 and 0.04, respectively).

As for DP domain of BOS, the possibility of getting high DP scores increased up to two times with the need for buy PPE from participants' own money, and with harassment by patients' families (OR = 2.12; *P* = 0.04 for both). While older age was associated with lower possibility of getting high DP score (OR = 0.91; *P* = 0.01), male sex and working in the ICU increased the likelihood of DP 2 times (OR = 2.07, *P* = 0.03), (OR = 2.16, *P* = 0.04), respectively (Supplementary Table 4).

In contrast to the other two domains of BOS; there was no impact of age, the need for buying PPE or harassment by patients' families on PA. Thus, the predictors of this domain were working in triage hospital and dissatisfaction about the cure rate from COVID-19. As shown in Supplementary Table 5, low PA scores were less likely to occur with working in triage hospital (OR = 0.35, *P* = 0.03), and with satisfaction about the cure rate of COVID-19 patients (OR = 0.27, *P* = 0.01).

## Discussion

Burnout syndrome is a state of physical and mental exhaustion related to work or caregiving activities (14, 15). Emotional exhaustion, depersonalization and diminished personal accomplishment are the main characteristics of this syndrome (11). While emotional exhaustion refers to feelings of overload and depletion of emotional resources; depersonalization represents a cynical “isolationist” attitude toward everyday interactions with others. Reduced personal accomplishment occurs when the subject feels less competent in his/her role (11, 16). With prevalence near to or exceeding 50%, BOS has become a serious mental health problem for healthcare professionals in many countries, including Egypt (7, 17–19). The consequences of BOS include increased risk of medical errors, decreased patient satisfaction, and depression (15, 17). COVID-19 pandemic is an exceptional situation that may add new factors to the development of BOS in healthcare workers, especially physicians. Here, we aimed at evaluating the frequency of burnout syndrome among a sample of Egyptian physicians during the COVID-19 pandemic and to identify some of its determinants. To the best of our knowledge, this is the first study that evaluates this frequency in Egypt during the pandemic.

Our results revealed that <40% of participants had BOS. While most of them suffered from reduced personal accomplishment, lower proportions of respondents scored high on depersonalization and emotional exhaustion. Our study population had low EE and DP scores (28.18, 31.82%, respectively compared to scores reported among Egyptian anesthesiologists (62.2%, 56.1, respectively) (20). Physicians and medical residents at the Suez Canal University Hospital (which serves the three Suez Canal-side cities as well as the Sinai Peninsula) reportedly had a higher prevalence of burnout when compared with our participants (53.9 vs. 36.36%) (21, 22). On the other hand, levels of burnout among our participants were higher than with that of physicians working in the emergency hospital of Tanta University and emergency medical responders in Mansoura City (19, 23). It is important to note that data collection was done during the peak of COVID-19 in Egypt, and these findings may change in the future. Therefore, we recommend repeating this study after the pandemic to assess the long-term effects of COIVD-19 on the development of BOS among Egyptian physicians.

In this survey, we examined several socio-demographic and job-related characteristics to understand the role of the COVID-19 in the development of burnout among our participants; different socio-demographic and job characteristics were examined. These factors could be reflected on the physical, psychological well-being and performance of the target group, and their identification could help personalize interventions (24). Younger age was associated with all BOS domains, and was a predictor of the development of BOS in general and of DP domain. This is similar to the findings in several studies, which showed that burnout levels in physicians tend to decrease with age (25, 26) These results make sense, since the work load is usually higher for younger physicians, which exposes them to excessive stress and higher risk of infection. This is usually accompanied by limited experience, especially while facing the “unknown” of the pandemic, which adds another factor to the pressure they face. Interestingly, while growth of experience was associated with higher PA scores among our group, dissatisfaction about the cure rate of COVID-19 patients was associated with lower PA scores. Moreover, satisfaction about the cure rate of patients was a predictor of high PA in our participants. Taken together, we think that although younger age is associated with development of BOS in our participants, witnessing the recovery of their patients increases their feelings of achievement, self-confidence and can provide some relief from the stress they face during COVID-19 era.

Long working hours and overnight shifts cause the medical profession to be among the most stressful jobs in Egypt for women. This stress may have an impact on their social life and family and may result in the development of fear and guilt. In our study, female physicians had higher EE scores in comparison to their male peers. Female physicians tend to be more empathetic with patients and try to take care of them at any cost (27). Moreover, female physicians tend to be more concerned about their families' well-being as a result of the pandemic. This result is consistent with previously published data about burnout among general practitioners (27). On the other hand, the male gender was found to be a predictor of DP, where the stress associated with the pandemic could lead to loss of motivation and a sense of isolation (28). We believe that gender differences should be considered while providing psychological support for physicians during the COVID-19 pandemic.

While professional aspects related to burnout are investigated, personal aspects should not be ignored. In our participants, infection of a colleague with COVID-19 was significantly correlated with higher EE score. Fears associated with the infection of colleagues exaggerate professional stress and the feeling of being emotionally worn-out and drained. If this is followed by death of a colleague or a relative due to COVID-19, this could be associated with the feeling that they are not competent enough to protect their family and friends, which is reflected in the form of lower PA. These are important new determinants of BOS that didn't exist before COVID-19 era.

Working in an intensive care unit (ICU) environment is known to be stressful. It is psychologically exhausting to be constantly dealing with seriously ill patients (29, 30). In our study, findings showed that participants working in COVID-19 isolation hospital ICUs had experienced higher degrees of EE. The stress associated with ICU work is amplified by the worries about the risk of infection or transmission of the COVID-19 virus to families or close relatives.

The economic burden of COVID-19 should not be overlooked. In a recent study, we showed that this was a major concern among the general public during the pandemic (31). In Egypt, physicians who work for the governmental healthcare system receive significantly lower salaries compared with those of physicians in western countries or the Gulf region. This is the case despite the long working hours and the possibility of exposure to infection, which may result in morbidity or even mortality (32).

An important finding in this study was that most participants (73.64%) reported using their private money to buy PPE. Although more than half of participants were satisfied with the availability of PPE in their hospitals, many of them had to buy PPE out of their own pockets. Different hospitals tried to provide medical personnel with basic PPEs. However, extra protective measures like using face shield masks and N95 masks were needed during the pandemic. Physicians in our study had to pay for them and look for other sources to purchase these supplies. It is not surprising that this factor was a predictor of BOS in general and for EE and DP domains. This is another determinant of BOS that didn't exist before the pandemic.

Higher EE was also associated with dissatisfaction with salaries. Many Egyptian physicians need to work in several healthcare facilities to achieve an acceptable income. Their ability to move between medical centers/hospitals was curtailed by the partial curfew that was imposed in Egypt during the height of the pandemic. Taken together, our study results indicate that the economic burden during the epidemic was a significant concern for our respondents, which would have affected their performance.

The lack of PPEs may have created a feeling among our participants the impression that the government and the community at large were not supportive or protective of healthcare workers. This may explain the high EE scores. More than three-quarters of participants in this study believed that a stigma had built up against health professionals dealing with COVID-19 in Egypt, and 40% of those dealing with COVID-19 patients reported exposure to harassment by families/relatives of patients. Some features of stigma were reported against medical physicians in Egypt. For example, incidents were reported where taxi drivers did not agree to drive medical physicians, and restaurants would refuse to deliver food to hospitals due to fear of infection with the COVID-19 virus (33). This explains their feelings of a lack of appreciation of their sacrifices by the society at large during the pandemic.

Although this harassment was not accompanied by reduced personal accomplishment, it led to higher EE and DP. Emotional exhaustion was apparently caused by physicians' fears that they or their families would be exposed to psychological or physical harm. Depersonalization could be a reaction to harassment and the perceived lack of appreciation by the community at large. Collectively, we think that this data represents a warning sign that explains why a large number of Egyptian physicians have sought for or plan to seek for work abroad to find better social and financial recognition. We think that the highest levels of financial and community support should be available for physicians during this pandemic. Infection control/prevention and personal protection training for these physicians is not less important. The latter can be implemented through virtual education and social media platforms, which have proven to be effective methods for communication and training in Egypt (31, 34).

There are some limitations of this study. First, this survey was conducted and administered through social networking sites, including Facebook and WhatsApp groups, and this may have resulted in sampling bias. Most participants were younger physicians. Younger age groups tend to be more social-media active and thus younger physicians were more likely to answer the survey. A second limitation was the relatively small sample size and the lacks of representation of various medical specialties. The questionnaire itself had some limitations as well. Since psychosocial factors are not included within the scale, we didn't ask about them. Factors related to working style, working environment, working conditions, workload, control, coworker support were not included in the study questions, either. However, our study sheds light for the first time on this serious topic, especially as the pandemic continues to claim new victims every day.

## Conclusions and Recommendations

Our findings show that the COVID-19 pandemic has added new demographic and job-related factors to the development of BOS among our research group of Egyptian physicians. From our perspective, several measures should be taken by the government and the society at large to support physicians at this stage. These measures include psychological and moral support, especially in different media platforms. Other measures should include setting better work hours, adjusting salaries, and providing supplies as well as infection prevention, PPE use and COVID-19 safety training.

## Data Availability Statement

The raw data supporting the conclusions of this article will be made available by the authors, without undue reservation.

## Ethics Statement

The studies involving human participants were reviewed and approved by The Ethics Committee of Alexandria University. The patients/participants provided their written informed consent to participate in this study.

## Author Contributions

ASA and AA designed the study, participated in data analysis, and manuscript writing. HHZ and ES participated in data collection and wrote the methods section. AMM participated in data collection and revised the manuscript. MA wrote the discussion section. All authors contributed to the article and approved the submitted version.

## Conflict of Interest

The authors declare that the research was conducted in the absence of any commercial or financial relationships that could be construed as a potential conflict of interest.
