# Supplementary material for: Prevalence, Associated Factors, and Consequences of Burnout Among Egyptian Physicians During COVID-19 Pandemic
Source: Front Public Health. 2020 Dec 3;8:590190. doi: 10.3389/fpubh.2020.590190 (PMC7744472; doi:10.3389/fpubh.2020.590190)
Supplement: Supplementary file 1 [file Data_Sheet_1.docx]

**Supplementary files**

**Supplementary table (1): Risk factors related to COVID-19**

| Risk factors | Total (n=220) | |
| --- | --- | --- |
| Do you currently deal with COVID-19 patients | **No** | **%** |
| No | 41 | 18.64 |
| Yes | 179 | 81.36 |
| If the answer is yes, where do you deal with them? * (n=179) |  |  |
| Isolation hospital | 78 | 35.45 |
| Isolation hospital ICU | 36 | 16.36 |
| Triage hospital (where cases are admitted temporarily for confirming diagnosis before being referred to the isolation hospitals) | 89 | 40.45 |
| Did you have to buy personal protective equipment from your own money? |  |  |
| No | 58 | 26.36 |
| Yes | 162 | 73.64 |
| Did you notice the mortality of patients from COVID-19 at your workplace?(n=179)** |  |  |
| No | 118 | 53.64 |
| Yes | 61 | 27.73 |
| Are you satisfied with the cure rate of COVID-19 patients? (n=179)** |  |  |
| No | 73 | 40.78 |
| Yes | 106 | 59.22 |
| Are you satisfied with the treatment protocol for COVID-19 patients? (n=179)** |  |  |
| No | 71 | 39.66 |
| Yes | 108 | 60.34 |
| Are you satisfied with the coordination between triage and isolation hospitals?(n=179)** |  |  |
| No | 116 | 64.80 |
| Yes | 63 | 35.20 |
| Have patients' families harassed you during your work with COVID-19 patients? (n=179)** |  |  |
| No | 106 | 59.22 |
| Yes | 73 | 40.78 |
| Have you been infected with COVID-19 during work? (n=197)** |  |  |
| No | 177 | 98.88 |
| Yes | 2 | 1.12 |
| Had any of your colleagues been infected with COVID-19? |  |  |
| No | 89 | 40.45 |
| Yes | 131 | 59.55 |
| Had any of your relatives been infected with COVID-19? |  |  |
| No | 212 | 96.36 |
| Yes | 8 | 3.64 |
| Has any of your colleagues or relatives died with COVID-19? |  |  |
| No | 210 | 95.45 |
| Yes | 10 | 4.55 |

* Some participants had more than one answer for this question

** Only physicians currently dealing with COVID-19 cases were included in these questions

**Supplementary table 2: Perceptions about the general situation related to COVID-19 in Egypt**

| Perception situation | Totally agree | | Agree | | Not sure | | Disagree | | Totally disagree | |
| --- | --- | --- | --- | --- | --- | --- | --- | --- | --- | --- |
|  | **N** | **%** | **N** | **%** | **N** | **%** | **N** | **%** | **N** | **%** |
| I feel satisfied with the presence of personal protective equipment where I work | **21** | **9.55** | **93** | **42.27** | **41** | **18.64** | **51** | **23.18** | **14** | **6.36** |
| I think there is an accepted level of awareness about the disease among the public | **4** | **1.82** | **32** | **14.55** | **53** | **24.09** | **105** | **47.73** | **26** | **11.82** |
| I think the general public appreciates the efforts of health professionals against COVID-19. | **10** | **4.55** | **49** | **22.27** | **78** | **35.45** | **68** | **30.91** | **15** | **6.82** |
| I feel stratified with the salary I get | **4** | **1.82** | **30** | **13.64** | **32** | **14.55** | **112** | **50.91** | **42** | **19.09** |
| I think there is stigma against patients with COVID-19 in Egypt | **104** | **47.27** | **74** | **33.64** | **25** | **11.36** | **16** | **7.27** | **1** | **0.45** |
| I think there is stigma against health professional dealing with COVID-19 in Egypt | **97** | **44.09** | **75** | **34.09** | **35** | **15.91** | **11** | **5** | **2** | **0.91** |

**Supplementary table 3: Predictors of emotional exhaustion (EE) domain of BOS among participants**

| Factors | OR | 95% CI | P |
| --- | --- | --- | --- |
| Age | 0.94 | (0.8726,1.0107) | 0.083 |
| Sex (male) | 0.61 | (0.3157,1.1725) | 0.14 |
| Marital status (single) | 1.13 | (0.4196,3.0641) | 0.80 |
| Having children (yes) | 1.52 | (0.5440,4.2350) | 0.42 |
| Comorbidity (yes) | 1.99 | (0.8978,4.3926) | 0.09 |
| Buy PPE from own money (yes) | 2.73 | (1.1434,6.4981) | 0.02 |
| Watching mortality from COVID-19 in workplace (yes) | 0.98 | (0.4552,2.1064) | 0.96 |
| Harassment by patients' families during work with COVID-19 Patients (yes) | 2.21 | (1.0485,4.6682) | 0.04 |
| Colleagues or relatives died from COVID-19 (yes) | 0.34 | (0.0601,1.8666) | 0.18 |

OR: odd ratio, CI: Confidence interval, P value considered significant if < 0.05

**Supplementary table 4: Predictors of depersonalization (DP) domains of BOS among participants**

| Factors | OR | 95% CI | P |
| --- | --- | --- | --- |
| Age | 0.91 | (0.8511,0.9822) | 0.01 |
| Sex (male) | 2.07 | (1.0835,3.9564) | 0.03 |
| Marital status (single) | 1.60 | (0.5968,4.2953) | 0.35 |
| Having children (yes) | 1.59 | (0.5796,4.3451) | 0.37 |
| Comorbidity (yes) | 1.48 | (0.6829,3.2161) | 0.32 |
| Working in isolation hospital ICU (yes) | 2.16 | (0.9767,4.7877) | 0.04 |
| Buy PPE from own money (yes) | 2.12 | (0.9742,4.6032) | 0.04 |
| Watching mortality from COVID-19 in workplace (yes) | 1.42 | (0.6988,2.9025) | 0.33 |
| Harassment by patients' families during work with COVID-19 Patients (yes) | 2.12 | (1.0168,4.4184) | 0.04 |
| Colleagues or relatives died from COVID-19 (yes) | 1.26 | (0.3057,5.1654) | 0.75 |

OR: odd ratio, CI: Confidence interval, P value considered significant if < 0.05

**Supplementary table 5: Predictors of personal accomplishment (PA) domain of BOS among participants**

| Factors | OR | 95% CI | P |
| --- | --- | --- | --- |
| Age | 0.96 | (0.8677,1.0556) | 0.39 |
| Sex (male) | 1.55 | (0.5751,4.1744) | 0.38 |
| Marital status (single) | 0.34 | (0.0776,1.4546) | 0.14 |
| Having children (yes) | 0.85 | (0.1813,3.9611) | 0.83 |
| Comorbidity (yes) | 0.57 | (0.1778,1.8150) | 0.35 |
| Working in triage hospital (yes) | 0.35 | (0.1387,0.9034) | 0.03 |
| Buy PPE from own money (yes) | 0.64 | (0.1951,2.1071) | 0.45 |
| Satisfaction with the cure rate of COVID-19 patients (yes) | 0.27 | (0.0949,0.7804) | 0.01 |
| Harassment by patients' families during work with COVID-19 Patients (yes) | 0.75 | (0.2525,2.2302) | 0.61 |

OR: odd ratio, CI: Confidence interval, P value considered significant if < 0.05
